# Ammoxidized Fenton-Activated Pine Kraft Lignin Accelerates Synthesis and Curing of Resole Resins

**DOI:** 10.3390/polym9020043

**Published:** 2017-01-28

**Authors:** Masoumeh Ghorbani, Johannes Konnerth, Enkhjargal Budjav, Ana Requejo Silva, Grigory Zinovyev, Hendrikus W. G. van Herwijnen, Matthias Edler, Thomas Griesser, Falk Liebner

**Affiliations:** 1 Department of Material Sciences and Process Engineering, Institute of Wood Technology and Renewable Materials; University of Natural Resources and Life Sciences Vienna, University and Research Center Tulln, Konrad-Lorenz-Strasse 24, 3430 Tulln an der Donau, Austria; marmar.ghorbani@boku.ac.at (M.G.); johannes.konnerth@boku.ac.at (J.K.); 2 Department of Chemistry, Division of Chemistry of Renewable Resources, University of Natural Resources and Life Sciences Vienna, University and Research Center Tulln, Konrad-Lorenz-Strasse 24, 3430 Tulln an der Donau, Austria; enkhjargal.budjav@boku.ac.at (E.B.); ana.requejo-silva@boku.ac.at (A.R.S.); grigory.zinovyev@boku.ac.at (G.Z.); 3 Kompetenzzentrum Holz GmbH, Altenberger Strasse 69, 4040 Linz, Austria; erik.van-herwijnen@boku.ac.at; 4 Chair of Chemistry of Polymeric Materials & Christian Doppler Laboratory for Functional and Polymer Based Ink-Jet Inks, University of Leoben, Otto-Glöckel-Strasse 2, A-8700 Leoben, Austria; matthias.edler@unileoben.ac.at (M.E.); thomas.griesser@unileoben.ac.at (T.G.)

**Keywords:** lignin, phenol formaldehyde resin, lignin ammoxidation, Fenton-type oxidation, ligneous adhesive, automated bonding evaluation system (ABES)

## Abstract

Ammoxidation of pine kraft lignin in aqueous 5 wt % ammonia affords a novel type of phenol substitute that significantly accelerates resole synthesis and curing as demonstrated for 40 wt % phenol replacement. Compared to non-ammoxidized lignin, which already shortens significantly the cooking time required to reach a resole viscosity of 1000 Pa·s (250 vs. 150 s) and reduces the typical curing B-time by about 25% at 100 °C, the use of ammoxidized lignin has an even more pronounced impact in this respect. Activation of lignin by Fenton-type oxidation prior to ammoxidation further boosts both synthesis and curing of the resole. This is presumably due to the intermediary formation of polyvalent cross-linkers like *N*,*N*,*N*-tris (methylol) trimethylene triamine triggered by saponification of a larger fraction of nitrogenous moieties present in such a treated lignin (ammonium salts, amide-type nitrogen, urea) and reaction of the released ammonia with formaldehyde. Except for the fact that phenol replacement by ammoxidized lignin results in a somewhat less brittle cured adhesive polymer and higher elastic modulus, the aforementioned acceleration in curing could no longer be observed in the presence of wood, where a significantly delayed wood-adhesive bond formation was observed for the lignin-containing adhesives as evident from the automated bonding evaluation system.

## 1. Introduction

Phenol formaldehyde (PF) resins are comparatively inexpensive commodity plastics and adhesives that feature good moisture, in addition to chemical and heat resistance, and have therefore found application in a vast variety of fields including engineered wood products, such as plywood, laminated veneer lumber, glue laminated timber, compact laminates or binders for mineral-based insulation boards [1,2]. The potential of lignin as the second-most abundant terrestrial biopolymer to replace phenol in the production of PF resins has been an intensively researched topic in material sciences for several decades [3,4,5,6]. The current upswing of renewables-based bio-economy approaches, the continually growing demand for phenolic resins, and the fact that technical lignins as a comparatively cheap by-product of wood pulping are still largely underutilized have further amplified these activities [2]. However, some properties inherent to lignin have hitherto prevented this bio-resource from entering many potential large-scale applications, including PF resins. This is mainly related to the irregular network polymer structure formed during lignin biosynthesis by enzyme-mediated radical dehydropolymerization of various phenolic precursor compounds. As the percentages of the principal lignin-forming building blocks vary to a large extent depending on the plant species, the macromolecular features of lignin follow this diversity as evident from the wide range of average molecular weight, branching, steric demand as well as pattern and abundance of functional groups [7]. These difficulties of working with lignin in material science are amplified by other factors including the different technical lignin isolation procedures which lead to comprehensive chemical alteration of the biopolymer in terms of de-polymerization; changes in functional group pattern; and even undesired re-polymerization in pulp production.

The reaction of formaldehyde with phenol in alkaline medium affording resole resins follows the principles of electrophilic aromatic substitutions with the phenolic hydroxyl groups directing the newly introduced hydroxymethyl (methylol) groups into the respective ortho and para positions. Subsequent non-linear step-reaction polymerization affords dense, rigid networks of phenolic moieties cross-linked by methylene, methylene ether and methylidene bridges whose percentages depend on the condensation conditions. Partial replacement of phenol by lignin in PF resins typically translates into a faster increase of viscosity during cooking, albeit with reduced mechanical properties of the cured lignin phenol formaldehyde (LPF) resin. While the rapid gain in viscosity is caused by the well-known chain-extending—or better network-expanding—effect of lignin, the inferior mechanical properties have been explained by formation of less dense and more irregular networks [6]. Even though the three principal phenolic moieties constituting the macromolecular structure of lignin (4-hydroxyphenyl, H; 4-hydroxy-3-methoxyphenyl, guaiacyl, G; 4-hydroxy-3,5-dimethoxyphenyl, syringyl, S) can react with formaldehyde in a similar manner, the specific substitution pattern of these building blocks considerably limits the number of aromatic ring positions available for methylolation. While the C3 side-chain in lignin generally blocks the p-position, the availability of the ortho positions largely depends on the plant species governing the H, G and S patterns. Softwood lignin, for example, consists of virtually only G units, whereas hardwood often contains G and S units in about equal portions; grass lignin is comprised of a wide mixture of all of them [7,8,9]. The availability of free phenolic groups is another factor that—independent of the degree of methoxylation—largely decides to what extent lignin can be methylolated as formation of phenolate ions is a prerequisite to electrophilic aromatic substitution by formaldehyde. Last but not least, it has to be taken into account that even if methylolation had occurred, steric hindrance can impede intermolecular condensation [6]. Several attempts have been made to improve the performance of lignins in PF synthesis, including pre-methylolation, demethylation to reduce the number of methoxyl groups in favor of phenolic hydroxyl groups, phenolation, hydrolysis, hydrogenolysis, oxidation and reduction [2,10,11,12]. However, none of these methods turned out to satisfactorily improve the reactivity of lignins for respective target applications [10].

Activation of lignin in terms of introduction of additional aliphatic and aromatic hydroxyl groups by Fenton oxidation has been successfully tested for cross-linking with oligo (alkylene glycol) diglycidyl ethers [13]. The term Fenton chemistry describes a complex set of reactions triggered by the joint action of hydrogen peroxide and catalytic amounts of iron (II) or other transition metal ions, such as Mn^2+^, Cu^2+^, Cr^2+^ or Co^2+^. Even though the mechanisms of the Fenton reaction are still under intense and controversial discussion, it has been proven beyond doubt that the Fenton reagent is a powerful oxidant of organic substrates regardless of whether a radical (HO• radical, one-electron oxidant) or non-radical mechanism (Fe^IV^O^2+^, two-electron oxidant) is assumed (for a short review see [14]). The effects of a Fenton-type treatment of lignin can be manifold due to its heterogeneous chemical composition including cleavage of β–O–1 and β–O–4 bonds [15], demethoxylation, aromatic and aliphatic hydroxylation [16,17], side-chain oxidation under the formation of aromatic aldehydes, ketones and carboxylic acids [15,18], aromatic ring cleavage, and condensation reactions [17]. Reduction of steric limitations by lignin depolymerization, along with the increase of functional group density, is considered beneficial for both homo- and copolymerization reactions.

Ammoxidation of lignin, i.e., its joint treatment with oxygen and ammonia [19], has been intensively studied for decades in an attempt to produce nitrogen-rich organo-mineral fertilizers featuring long-lasting nitrogen release (high-pressure approaches; up to 25% N) or artificial humid substances featuring C/N ratios typical for humified soil organic matter (10–15) and moderate nitrogen contents bound in different types of nitrogenous moieties which mineralize in soil at different rates (ambient-pressure approaches; ca. 5%–6% N) [20]. The conducted studies have shown that—depending on reaction conditions—ammoxidation of lignin involves a multitude of simultaneously occurring reaction sequences which include oxidation, demethylation, demethoxylation, depolymerization of lignin, cleavage of aromatic rings and formation of muconic acid derivatives. The variety of degradation products and newly introduced functional groups results in a broad spectrum of nitrogen bonding types including ammonia salts, urea, amides, amino quinones, or nitrogen bonded in heterocyclic structures [21]. The existence of the different nitrogenous compounds and moieties has been hypothesized to be beneficial for the synthesis of LPF resole resins, as either low-molecular nitrogenous ammoxidation products, such as urea and amino benzoquinones, or ammonia released by alkaline saponification which can react with formaldehyde to methylolated intermediates that can supposedly act as cross-linkers during LPF synthesis and curing. Addition of urea affording phenol-urea-formaldehyde resins, for example, has shown to accelerate cooking of the resin considerably [22], presumably due to the cross-linking activity of methylolated urea species. Ammonia can react with formaldehyde in a similar way. It is known that sequential saponification of ammoxidized lignin according to Wang et al. [23] and Schiene et al. [24] using hot aqueous Mg(OH)_2_ and NaOH, respectively, releases ammonia from about 50% of the total nitrogen incorporated into lignin during ammoxidation. This should be also the case during cooking of resole resins due to the strongly alkaline medium and high temperature (≤80 °C). The released ammonia competes with phenol and lignin for formaldehyde. With the latter, ammonia forms in aqueous alkaline medium methylolated amines, such as *N*,*N*,*N*-tris (methylol) trimethylene triamine, which can act as cross-linking agents during resole cooking and curing.

From an economic perspective, Fenton activation and subsequent ammoxidation is an uncomplicated, inexpensive approach as both steps can be conducted successively in aqueous alkaline medium. While Fenton-type oxidation is conducted with comparatively low concentrations of the oxidant H_2_O_2_ (≤3%) and catalytic amounts of iron II salts only, aqueous ammonia (5%) and finely dispersed air are the two media required for the ammoxidation process.

In this study, the impact of Fenton-type activation and ammoxidation of lignin on the cooking behaviour and adhesive properties of LPF resins was studied. Lignin-free PF resole resins prepared at common conditions were compared with the respective LPF analogous materials that were obtained by replacing 40 wt % of phenol by (1) unmodified; (2) ammoxidized and (3) Fenton-oxidized and subsequently ammoxidized pine kraft lignin. Pine kraft lignin has been chosen as a substrate since it has recently been shown to (A) feature the highest reactivity towards electrophilic aromatic substitution by formaldehyde [8,9], and (B) have the best adhesive properties among various types of tested lignins (grass soda lignin, softwood organosolv lignin, pine kraft lignin or sodium lignosulfonate) [6]. Organosolv lignin is supposedly less suited for replacing phenol in resole resins as the abundance of free, non-etherified phenolic hydroxyl groups is rather low due to the specific process conditions. Lignosulfonates, on the other hand, comprise a significantly more polar and hydrophilic group compared to other lignins which would counteract the good moisture-resistance of PF resins. The impact of the different lignin modifications on the extent of structural alterations were investigated by elemental analysis, calculation of aromaticity using the modified Rentrop function, size exclusion chromatography, and functional group analysis including ^1^H, ^31^P-NMR and X-ray photoelectron spectroscopy. The PF network-expanding effect of the lignins and the extent of methylolation during resole cooking were monitored by viscosimetry and formaldehyde consumption. Thermal and mechanical properties of the obtained adhesives were evaluated based on the time to reach the B-stage of curing (B-time), differential scanning calorimetry (DSC), tensile shear strength development of adhesive bonds as a function of hot pressing time using an automated bonding evaluation system (ABES), and nanoindentation of cured adhesive polymers.

## 2. Materials and Methods

### 2.1. Materials

Pine kraft lignin Indulin AT^TM^ was purchased from Mead Westvaco Corp (Richmond, VA, USA). The starting material had an ash content of 3.0%, Klason lignin content of 91.7%. Phenol (≥99.5%), formaldehyde (Formalin, 37% aqueous solution), distilled water, hydroxylamine hydrochloride, sodium hydroxide solution (1 mol/L) and hydrochloric acid (1 mol/L) and isopropanol were purchased from Carl Roth GmbH & Co. KG (Karlsruhe, Germany). Sodium hydroxide (97%), dimethyl sulfoxide (DMSO), lithium bromide (LiBr), pyridine, cholesterol and *N*-hydroxy-5-norbornene-2,3-dicarboxylic acid imide (e-HNDI) were obtained from Sigma-Aldrich Co. LCC (Steinheim, Germany). Hydrogen peroxide was supplied by Panreac Quimica SLU (Barcelona, Spain). Iron(II) sulfate heptahydrate (FeSO_4_·7H_2_O) was purchased from Merck GmbH (Vienna, Austria), sulfuric acid (95%–97%) and aqueous ammonia (28% *v/v*) from VWR International (Vienna, Austria). DMSO-d6 and CDCl_3_ were obtained from Euroiso-top (Saint-Aubin, France), and 2-Chloro-4,4,5,5-tetramethyl-1,3,2-dioxaphospholane (TMDP) was bought from ChiroBlock Inc. (Wolfen, Germany). Chromium (lll) acetylacetonate was purchased from Honeywell Fluka (Bucharest, Romania).

### 2.2. Methods

#### 2.2.1. Lignin Modification (cf. Table 1)

##### Ammoxidation of Lignin

A solution of 15 g PK lignin in 350 mL aqueous ammonia (5%) was placed in a 600 mL pressure vessel (Parr Instruments, 4566 C Series Frankfurt, Germany), equipped with heating jacket and temperature controller. The reactor was pressurized with O_2_ to 0.2 MPa and heated up to 70 °C for 4 h under continuous stirring. After cooling, the reaction mixture (APK lignin) was transferred to a Petri dish, then consecutively air- (25 °C) and vacuum-dried (40 °C) for 24 h each.

##### Fenton-Type Activation and Subsequent Ammoxidation of Lignin

The introduction of hydroxyl groups by Fenton-type oxidation was accomplished by hydrogen peroxide treatment of PK lignin dissolved in aqueous alkaline medium as described elsewhere [13]. In brief, 5 g PK lignin was dissolved under stirring in 20 mL of 0.2 M aqueous NaOH at room temperature, affording a 25% (*w/v*) solution. Then 0.1 mmol (109 mg) FeSO_4_·7H_2_O was added per gram of lignin and the mixture was stirred for another 30 min. Aqueous H_2_O_2_ (5%, *v/v*) was added drop by drop to adjust a concentration of 1.5% *v/v* hydrogen peroxide. After stirring for 24 h at room temperature, the oxidized lignin was precipitated by adding concentrated sulfuric acid until pH 2 was reached. After filtration, the crude product was washed with distilled water and dried in a vacuum oven at 40 °C for 24 h. Ammoxidation of the Fenton-oxidized PK lignin was accomplished in the same way as described in Section 2.2.1.

#### 2.2.2. Lignin Characterization

Elemental Analysis was performed on a Eurovector EA 3000 Elemental Analyzer (EuroVector S.p.A., Milan, Italy).

^1^H-NMR spectra were recorded on a Bruker Avance II 400 (resonance frequencies 400.13 MHz for ^1^H) equipped with a 5 mm broadband probe head (BBFO). Standard Bruker pulse programs were used. DMSO-d6 was used as solvent.

Quantitative ^31^P-NMR analysis of the different types of hydroxyl groups present in lignin was performed according to a procedure reported elsewhere [25] using 2-chloro-4,4,5,5-tetramethyl-1,3,2-dioxaphospholane (TMDP) as derivatization reagent. In brief, 25–30 mg lignin was dissolved in 700 μL of a 1:1.6 (*v/v* ratio) mixture of dry CDCl_3_ and pyridine). A total of 100 μL of standard stock solution containing cholesterol (50 mg/mL) and *N*-hydroxy-5-norbornene-2,3-dicarboxylic acid imide (e-HNDI) (30 mg/mL) and 100 µL of a solution containing the relaxation reagent chromium (lll) acetylacetonate (5 mg/mL) were added. After thorough mixing, 100 μL of the phosphitylation reagent were finally added. The total amount in the vials was 1 mL. The derivatization mixture was sealed and shaken for 1 h at room temperature and then transferred into an NMR tube. NMR spectra were recorded on a Bruker Advance II 400 equipped with a 5 mm broadband probe head (BBFO). Standard Bruker pulse programs were used. Peak assignment and quantitative calculation were performed as described elsewhere [26,27].

Molar mass analyses were performed using a Dionex Ultimate 3000 system (Thermo Fisher Scientific, Darmstadt, Germany) comprising of an autosampler, degasser, column oven, UV detector, and refractive index (RI) detector (Shodex RI-101, Munich, Germany). Separation was achieved by connecting three Agilent Polar-Gel M columns (7.5 mm × 300 mm) in a series which were calibrated by narrowly distributed polystyrene sulfonate standards (PSS) of known molecular mass: *M*_w_ = 1100, 1920, 3610, 6520, 14,900, 29,100, 63,900, and 14,8000 g·mol^−1^, *D* ≤ 1.20. Dimethyl sulfoxide (DMSO) containing LiBr (0.5% *w/v*) filtered through a 0.2 µm filter was used as the mobile phase. Samples were prepared by dissolution of 10 mg in 1 mL of DMSO/LiBr (0.5% *w/v*) and subsequent filtration through a 0.45 µm PTFE filter. Separation and detection conditions were as follows: flow rate 0.5 mL·min^−1^; column temperature 40 °C; injection volume 10 µL; run time 65 min; and UV detection at 280 nm (35 °C). Data evaluation was performed using Chromeleon software, version 6.80.

The monosaccharides constituting the hemicellulose fraction has been determined by acid methanolysis followed by gas chromatography [28].

XPS spectra were recorded using a Thermo Fisher Scientific, East (Grinstead, UK) instrument equipped with a monochromatic Al K-Alpha X-ray source (1486.6 eV). Element scans were acquired at a pass energy of 50 eV and a step size (resolution) of 0.1 eV. Wide scans were acquired with pass energy of 100 eV and a step size of 1.0 eV. All spectra have been normalized to the Au 4f7/2 peak. Charge compensation was performed with an argon flood gun. The average chemical composition was calculated from wide scan spectra in two different locations on each surface. The peaks were fitted using a Gaussian/Lorentzian mixed function employing a Shirley background correction (Software Thermo Avantage v5.906).

#### 2.2.3. Resin Preparation

##### Preparation of Phenol-Formaldehyde (PF) Resin

PF resole resins were synthesized as described elsewhere [6]. In brief, phenol and aqueous sodium hydroxide (50 wt %) were placed and heated in a 500 mL three-necked flask equipped with a condenser, electronic temperature controller and Overhead Stirrer Impellers—Blade and Half-Moon Styles (Heidolph, Germany). When the mixture had reached 65 °C, aqueous formaldehyde (37 wt %) was added at a flow rate of 8.43 mL·min^−1^ using an automatic burette (TitroLine6000/7000^®^, SI Analytics, Mainz, Germany). Then, the temperature was increased to 80 °C. When the viscosity had reached about 600 mPa∙s, the temperature was reduced to 70 °C to slow down the rate of viscosity increase. Cooking was stopped at a viscosity of approximately 1000 mPa∙s by rapid cooling using an ice bath.

##### Preparation of Lignin-Phenol-Formaldehyde (LPF) Resin

LPF resole resins of a phenol-by-lignin replacement level of 40 wt % were prepared according to the above procedure and have been detailed in previous work [6]. Deviating from PF synthesis, lignin was added portion-wise to the alkaline mixture of phenol and sodium hydroxide at 65 °C.

#### 2.2.4. Resin Characterization

Polymerization progress was monitored measuring the viscosity of cooled (20 °C) aliquots (1.1 mL) of the reaction mixture according to the German DIN 16916-2 standard [29]. A cone-plate rheometer (Bohlin CVO; Malvern Instruments Limited, Malvern, UK) equipped with a temperature control unit was used.

The solid content of the resins was determined according to ISO 3251 standard [30]. All samples were initially cured and equilibrated in a ventilated oven at 135 °C for 24 h, as recommended for liquid phenolic resins.

The free formaldehyde content was determined according to the ISO 11402 [31] by a reaction with hydroxylamine hydrochloride and back-titration of the released HCl using 1 M NaOH. In brief, a well-defined amount of resin (5.0 ± 0.2 mg) was dissolved in 50 mL of a mixture consisting of isopropanol and water (3:1 *v/v*). The pH of this mixture was adjusted to 3.5 with 1 M hydrochloric acid using a TitroLine^®^ 6000/7000 titrator (SI Analytics, Mainz, Germany). Then, 25 mL of hydroxylamine hydrochloride solution (10 wt %) were added under continuous stirring. After a reaction of 10 min, the solution was titrated to pH 3.5 using 1 M aqueous sodium hydroxide.

The time required to reach the B-stage of resin curing (B-time) was measured according to DIN 16916 [29] using self-constructed equipment. The latter consisted of a heated aluminum plate with a depression (diameter 25 mm, depth 5 mm) on it and a Pt100 temperature sensor for precise temperature control. Aliquots (500 mg) of the PF and LPF resins, respectively, were placed inside the depression at a plate temperature of 100 °C which was controlled at an accuracy of ±0.5 °C. A glass rod was used to stir the sample for one minute. After that, samples were stirred every minute for 10 s until the B-stage was reached. This was the case when the resin sample was no longer stringy and could be torn off at the end of the glass rod while it was pulled out. The test was performed at 100 °C instead of 130 °C as suggested in the standard procedure as such high temperatures are hardly achievable in solid wood bonding.

For Differential Scanning Calorimetry (DSC), approximately 10 mg of adhesive were placed into a 30 µL high-pressure steel crucible, equipped with gold sealing. A DSC 200 F3 Maia^®^ differential scanning calorimeter (Erich Netzsch GmbH & Co. Holding KG, Selb, Germany) was used to perform thermal analysis. All curing experiments were run at heating rates of 5 °C·min^−1^ in a temperature range of 20–250 °C. Thermograms were recorded using the Netzsch Proteus^®^ 4.8.2 software package (Selb, Germany). For each resin, two measurements were conducted.

A self-constructed ABES analogue device capable of evaluating the development of bonding strength during hot pressing of resin joints [32] was mounted onto a Zwick/Roell Z100 universal testing machine (Zwick GmbH & Co. KG, Ulm, Germany) using a controlled hot press temperature of 120 °C [33,34]. To provide the lap joints, two beech veneer strips of 0.58 mm thickness, 20 mm width, and 147 mm length stored at 20 °C and 65% relative humidity were glued together with an overlap length of 4 mm using a spread rate of 200 g·m^−2^ and a relative pressure of 1.36 N∙mm^−2^ [35]. Tensile shear strength was measured in a hot state immediately after the pressing time of 30, 60, 90, 120, 240, 360, 420, 480, or 720 s had elapsed. For each bond formed within a certain hot pressing time, eight specimens were tested.

**Figure 1:**
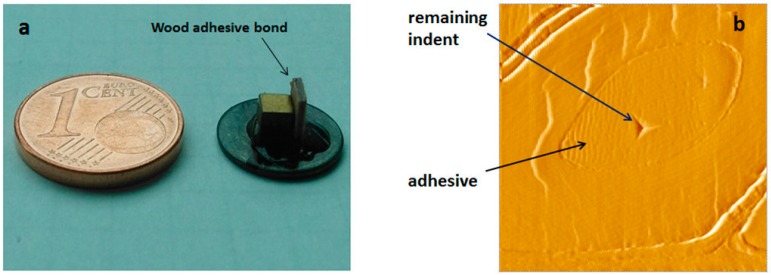
Nanoindentation sample of a few millimetres’ edge length, cut off from the glued beech veneers (**a**); Scanning probe microscopy image illustrating a nanoindent measurement point in an adhesive-filled cell lumen, scan size 15 µm × 15 µm (**b**).

The mechanical characterization of cured adhesive polymer present in wood-adhesive bonds was evaluated by means of nanoindentation [36,37,38]. The specimens were prepared from two beech veneers as they were used for the ABES test employing a hot pressing time of 12 min. Other parametres were kept similar to those described in the ABES section above. For each resin, two tiny pieces of a few millimetres’ edge length were cut off from the glued beech veneers (Figure 1a). Smooth surface was prepared by ultra-microtoming (Ultracut R, Leica, Austria) with a histo diamond knife (Diatome, Switzerland). The measurement points were selected from adhesive present in cell lumen as shown in the example of Figure 1b. For each piece, 10 points were tested. The nanoindentation experiments were performed with a Hysitron TriboIndenter system (Hysitron, Inc., Minneapolis, MN, USA) equipped with a three-sided pyramidal diamond indenter tip (Berkovich type). Proper positioning of each individual indent was controlled by using the scanning probe microscopy mode of the device. The reduced elastic modulus (Er), hardness (H) and indentation creep (C_IT_) were obtained from the load-depth curve and the related equations as described elsewhere [37]. Load curves were obtained for 3 s loading (500 µN), 20 s holding and 3 s unloading.

## 3. Results and Discussion

Three different types of ligneous resole resins containing pine kraft lignin (Indulin AT^TM^), in differently modified forms to substitute 40 wt % of phenol, were prepared and characterized for their adhesive properties: (A) PF resin containing unmodified pine kraft lignin; (B) PF resin containing ammoxidized pine kraft lignin; and (C) PF resin containing Fenton-oxidized and subsequently ammoxidized pine kraft lignin.

Size exclusion chromatography confirmed a pronounced increase of the molecular weight average (Table 2) of the studied lignin caused by its joint treatment with oxygen and ammonia in aqueous medium (ammoxidation). This is due to the well-known oxidative depolymerization of lignin in alkaline medium and subsequent condensation of intermediary, low molecular, highly reactive phenolic and quinoid compounds largely governed by ammonia [39]. As a result, lignin is considerably enriched with nitrogen which exists in various types of nitrogenous moieties including ammonium, aminoquinones, urea, amides, imides, and hetero-aromatic structures. According to elemental analysis, the amount of nitrogen in the studied pine kraft lignin (APK lignin) increased considerably from 1.17 to 6.06 wt % after ammoxidation (Table 3).

Fenton-type oxidation of lignin that had been expected to further activate lignin by enforced cleavage of intra- and interlignol bonds, as well as introduction of hydroxyl groups, did not afford ammoxidized products of further enhanced nitrogen content (Table 3), but resulted in a significantly lower weight average molecular weight (ca. 6500 Da) compared to the PK sample that was directly subjected to ammoxidation (ca. 14,000 Da; APK; Table 2). Considering the otherwise identical reaction conditions the obtained results suggest and confirm a stronger degradation of lignin mediated by the Fenton reaction compared to common oxidation in aqueous alkaline medium which, however, does not necessarily translate into more pronounced follow-up reactions with ammonia and nitrogen enrichment as evident from elemental analysis (Table 3).

Elemental analysis furthermore shows that for FAPK the H/C atomic ratio (1.205) as an indicator of condensation and aromaticity, respectively [40], is intermediary between that of the parent kraft lignin (1.145) and the respective product obtained from the parent lignin by direct ammoxidation without prior Fenton-type oxidation (1.244). The same trend has been confirmed by ^1^H-NMR spectroscopy as the integral ratio of protons attached to aromatic and aliphatic moieties changes in the same order, i.e., PK (1.27) < FAPK (1.39) < APK (1.65). The impact of Fenton-type oxidation and ammoxidation has been also investigated using the Rentrop function that calculates a Ring-Double bond Equivalent (RDE) sum parameter from atomic ratios and reports the content of rings and double bonds without revealing their exact number (Equation (1)).

(1) $$f_{a}=2-\frac{H}{C}+\frac{N}{C}-e^{-0.88\frac{H}{C}}-\left(0.58-0.55\frac{O}{C}\right)\frac{O}{C}$$

(2) $$f_{a}=1.8471+\frac{N}{C}-\left(\frac{H}{C}+e^{-0.88\frac{H}{C}}\right)+0.55{\left(\frac{O}{C}-0.5273\right)}^{2}$$

Application of this method—originally proposed to estimate the degree of coalification (aromaticity index) [41]—confirmed the consistency of all conclusions drawn from the results of SEC, elemental analysis, and ^1^H-NMR spectroscopy as the *f*_a_ values increased in the order PK (0.852) < FAPK (0.943) ≤ APK (0.945). According to the modified Rentrop function separating the effect of the different atomic ratios [40] (Equation (2)), both increasing H/C and N/C atomic ratios translate into higher *f*_a_ values and hence higher degrees of condensation. This applies for the parabolic O/C dependency as well, as O/C values increasing up to the apex of this function (O/C = 0.527) also lead to increasing degrees of aromaticity.

**Figure 2:**
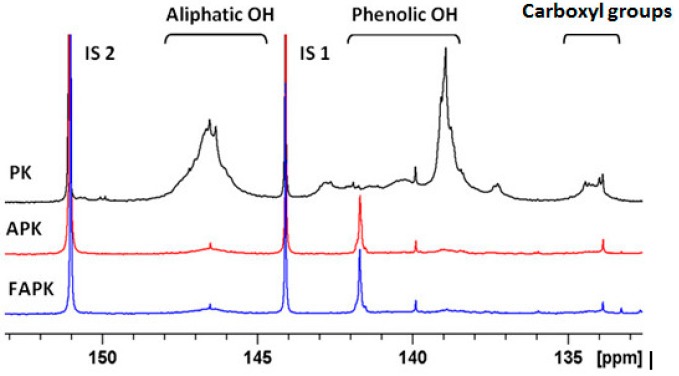
^31^P-NMR spectra of phosphitylated pine kraft lignin (PK) and pine kraft lignin derivatives (APK: ammoxidized PK; FAPK: Fenton-oxidized and subsequently ammoxidized PK).

Quantification of aliphatic and aromatic hydroxyl groups by ^31^P-NMR spectroscopy after derivatization of the samples with 2-chloro-4,4,5,5-tetramethyl-1,3,2-dioxaphospholane (Figure 2) confirmed that direct ammoxidation of the parent lignin affords both a higher degree of condensation and molecular weight compared to the Fenton-oxidized material. This is evident from the decreasing amount of aliphatic, phenolic and total hydroxyl groups (PK > FAPK > APK; Table 4).

**Figure 3:**
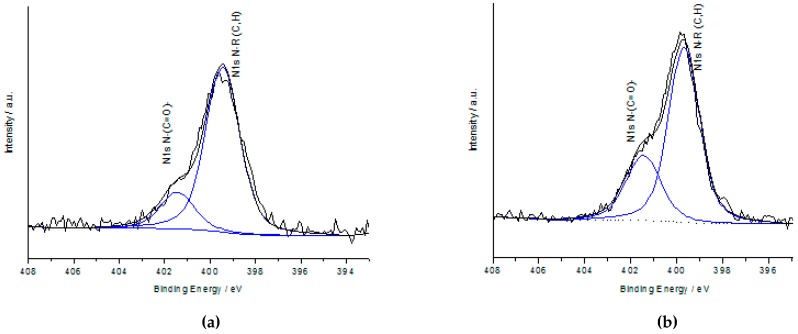
XPS spectra (N1s scans) of FAPK (**a**) Fenton-oxidized and ammoxidized PK and APK (**b**) ammoxidized pine kraft lignin.

X-ray photoelectron spectroscopy confirmed the nitrogen enrichment for both of the ammoxidized samples APK and FAPK (Figure 3). However, distinct differences can be seen for the contribution of the different nitrogenous moieties peaking at 399.7 eV (ammonium, amine) and 401.5 eV (amide). While APK contains a considerable quantity of amide-type nitrogen, FAPK is rather deficient in amides but has much more ammonium acting as counter-ions of carboxyl groups. Furthermore, XP spectra clearly confirm the higher degree of condensation for APK compared to its Fenton-oxidized and ammoxidized counterpart FAPK as evident from the significantly reduced peak at a binding energy of 286.5 eV (C1s) assigned to C–O–C and C–OH moieties, respectively. Both the C1 and N1 scans show that APK contains almost twice as many carboxyl moieties than FAPK, as well as nitrogen bound in amide-type structures, thereby suggesting the formation of muconic acid derivatives as proposed elsewhere [42] (Table 4).

**Figure 4:**
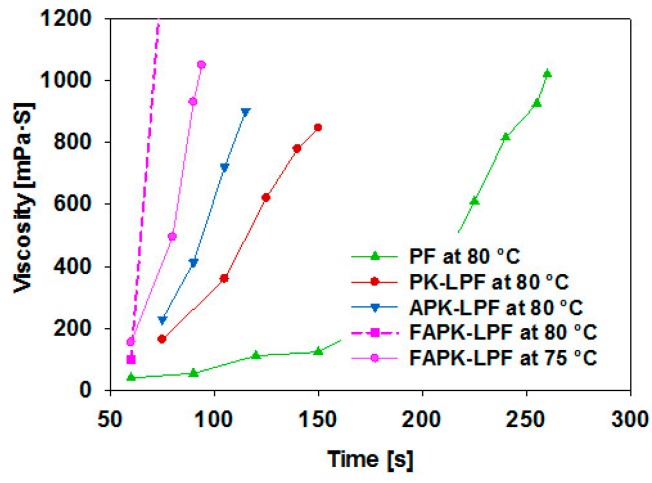
Viscosity development during cooking of the different resins; LPF resins were prepared substituting 40 wt % phenol by pine kraft (PK) lignin, ammoxidized pine kraft (APK) and Fenton-oxidized plus ammoxidized pine kraft (FAPK) lignin.

It has been shown in a previous study that replacement of phenol by 40 wt % of pine kraft lignin accelerates considerably the resole cooking process as evident from viscosity development [6]. This has been confirmed by this study where both the lignin-free and the 40 wt % lignin-containing PF resole resins have been used as reference materials (Figure 4). Replacement of pine kraft lignin by the same quantity of ammoxidized pine kraft lignin was now shown to have an even more pronounced effect in this respect. One might be tempted to explain that with the higher average molecular weight of APK that has clearly a network-expanding effect to the PF resin. However, the rate of viscosity increase is obviously governed by other effects as well, as Fenton-oxidized and subsequently ammoxidized lignin has an even more pronounced impact on viscosity development even though its molecular weight is only half that of APK. Formaldehyde addition and condensation were here so fast that the target viscosity of 1000 mPa∙s was unattainable for FAPK (Figure 4). Therefore, we reduced the cooking temperature to 75 °C to get better control of the viscosity development. The fast viscosity increase observed for FAPK is possibly the result of the elevated contents of ammonium salts present in Fenton-oxidized and subsequently ammoxidized pine kraft lignin (FAPK) as the comparatively strong alkaline resole cooking conditions can be assumed to afford significant amounts of ammonia which reacts in the presence of an excess of formaldehyde via the intermediates, methylene imine and trimethylene triamine, to *N*,*N*,*N*-tris(methylol) trimethylene triamine as described elsewhere [43,44]. The latter, in turn, could act as potent cross-linker connecting up to three methylolated phenol and lignin molecules which would explain the rapid viscosity gain of the formed FAPK resole resin. For APK, the lower amount of ammonia groups present in directly ammoxidized kraft lignin translates into reduced cross-linking by *N*,*N*,*N*-tris(methylol) trimethylene triamine which cannot be compensated by the higher molecular weight of ammoxidized lignin. Further factors supposed to have a minor impact on curing speed and bonding behaviour of wood composite, such as pH, solid content and free formaldehyde content are compiled in Table 5. It is evident that the different ammoxidation approaches had no significant influence on the alkalinity of the respective LPF resins. The solid contents of all resoles were quite similar as they were adjusted by adding small amounts of water to the readymade resins. Free formaldehyde content of the resins—used here as an indicator of formaldehyde consumption [6]—dropped from PK-LPF (9.2 wt %) to APK-LPF (8.1 wt %) by about 12%, reflecting the observed differences in viscosity development. Surprisingly, the free formaldehyde content of the fastest polymerizing resin (FAPK-LPF) was intermediary only between that of APK-LPF and PK-LPF (8.8 wt %), possibly due to release of formaldehyde during cross-linking of FAPK with *N*,*N*,*N*-tris (methylol) trimethylene triamine.

The reactivity of the prepared PF (pre-polymer) resins with regard to further curing has been evaluated by B-time testing according to DIN 16916 [29]. Even though being somewhat operator-dependent, this method is considered to be sufficiently reproducible and applicable to many phenolic resins [45] to investigate their curing speed at a given temperature (100 °C in this study). It is the time elapsed after which resins of known concentration change to the B-state when linear growth and branching of monomers and oligomers fade out. For the PF resin, a B-time of 7 min 38 s was determined, whereby all LPF resins had lower values (Table 6). The lowest B-time value was observed for FAPK-LPF resins indicative of a comparatively high reactivity which is assumed to be due to both the comparatively high free formaldehyde content (cf. Table 5) and the supposedly higher amount of remaining methylol groups introduced by reaction of FAPK with *N*,*N*,*N*-tris(methylol) trimethylene triamine.

**Figure 5:**
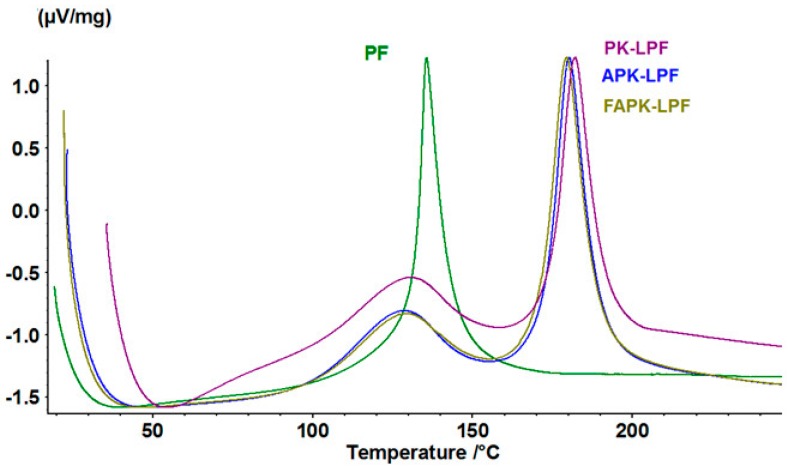
Differential scanning calorimetry (DSC) curves (heating rate 5 °C·min^−1^) of the reference (PF) and the LPF resins prepared from pine kraft (PK), ammoxidized pine kraft (APK) and Fenton-oxidized plus subsequently ammoxidized pine kraft (FAPK) lignins.

The curing behaviour of all resins was also evaluated using differential scanning calorimetry (DSC) at a heating rate of 5 °C·min^−1^ (Figure 5). It is evident that replacement of 40 wt % phenol by lignin—independent of its type of modification—translates into a significantly different curing behaviour. While a single, comparatively sharp exothermic event peaking at about 136 °C is characteristic for lignin-free resoles, two peaks were obtained for all lignin-containing resins. According to the peak temperature of lignin-free PF resoles (ca. 136 °C), the studied phenol-by-lignin replacement level of 40 wt % and the contribution of the two LPF peaks to the total heat evolved during curing (ca. 62%–64% at about 130 °C, ca. 36%–38% at about 180%; cf. Table 7). It can thus be concluded that the peak at about 180 °C is caused by curing reactions involving reaction centres in lignin-rich moieties poorly accessible due to steric hindrance and requiring sufficiently high activation energy. Curing of phenol-rich domains, on the contrary, occurs in a similar temperature range as for PF resins. However, the range is here much wider (55–155 °C) and starts at a comparatively low temperature due to involvement of activated phenolic moieties present in (modified) pine kraft lignin. Comparing APK-PF and FAPK-PF resins, the exothermic peak temperature for FAPK-PF resin was slightly lower. The onset temperature was 177.2 °C for PK-LPF resin, whereas it decreases to 173.6 °C (Δ3.6 °C) and 171.7 °C (Δ5.5 °C) for APK-LPF and FAPK-LPF resins, respectively. Simultaneously, the peak and end set temperatures decreased but to a somewhat lower extent. The peak temperature of PK-LPF resin which was 182.1 °C dropped to 180.2 °C (Δ1.9 °C) and 179.3 °C (Δ2.8 °C) for APK-LPF and FAPK-LPF, respectively. The fact that the most pronounced shift of the exothermic curing peak towards lower temperature was observed for the FAPK-LPF resin is in good agreement with the short B-time and the fast viscosity increase for this type of resin. It further supports the above assumption that stronger lignin depolymerization, less steric hindrance, higher ammonium contents and extent of LPF cross-linking mediated by *N*,*N*,*N*-tris(methylol) trimethylene triamine could be important factors that render FAPK-LPF more reactive compared to APK-LPF or PK-LPF.

**Figure 6:**
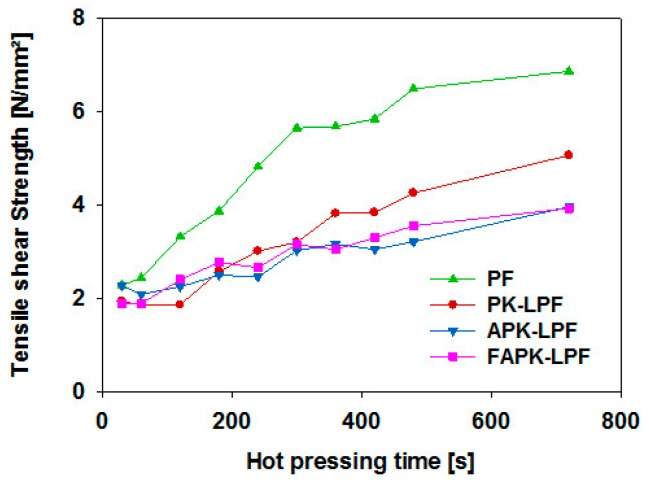
Tensile shear strength development as investigated by automated bonding evaluation system (ABES) at 120 °C hot pressing temperature as a function of pressing time.

The performance of the adhesives in the presence of adherents can be evaluated by monitoring the tensile shear strength development tested immediately after different times of hot pressing. This can be examined using the ABES technique which examines the potential of adhesives to be used for end products where processing time is crucial. Besides hot pressing, temperature and time, chemical properties of the adhesive and adherent materials are key factors governing bonding strength development [46]. The ABES results obtained in this study suggest that for the actual hot pressing conditions (120 °C, 1.36 N·mm^−2^, *t*_max_ = 760 s) replacement of 40 wt % phenol by pine kraft lignin (PK-LPF) slows the bonding process down. This was also the case for the two resins prepared from the ammoxidized lignins (APK-LPF and FAPK-LPF; Figure 6). This is in agreement with previous work where we demonstrated that only up to a phenol replacement level of about 20 wt % the bonding strength development of the comparable lignin-free PF resin can be retained while higher lignin contents have a negative impact on bond strength development [6]. This study also revealed that the inferior bonding strength development of lignin-rich PF resins translates into reduced ultimate bond strength measured immediately after long hot pressing as demonstrated for the selected pressing conditions (*T* = 120 °C, *t*_max_ = 760 s, *p*_rel_ = 1.36 N·mm^−2^). While the tensile shear strength of the reference PF resin was as high as 6.86 N·mm^−2^, the lignin-containing PK-LPF resin reached 5.06 N·mm^−2^ only. Phenol replacement by ammoxidized lignin surprisingly translated into further reduced ultimate strength (APK-LPF: 3.96 N·mm^−2^; FAPK-LPF: 3.93 N·mm^−2^) which contrasts the results of differential scanning calorimetry and B-time measurements at a first glance but could be explained by more rapid network formation due to the significantly higher molecular weights of both types of ammoxidized lignins at the expense of dense cross-linking. It has to be considered that tensile shear strength is measured immediately after hot pressing at an elevated temperature when the ABES technique is used. The lower absolute strength values for the lignin-containing adhesives may therefore also originate from a postulated higher temperature dependency of the LPF adhesives. As tensile shear strength of cured specimens of PF-bonded solid wood lap-joints tested at room temperature were observed to perform equally to those made out of PK-LPF after one week of storage in controlled climate [6], it is concluded that the lower ultimate bond strength measured by ABES does not imply a low final product performance.

**Figure 7:**
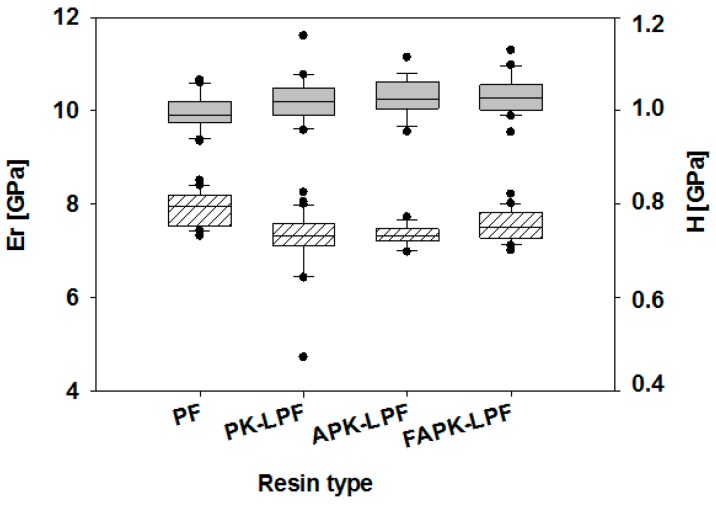
Results of nano-indentation testing: reduced elastic modulus (Er) (grey boxes) and hardness (H) (striped boxes) of cured adhesive present in bonds prepared from the lignin-free reference material (PF) and resole resins containing non-modified (PK-LPF) and differently ammoxidized pine kraft lignins (APK-LPF, FAPK-LPF).

**Figure 8:**
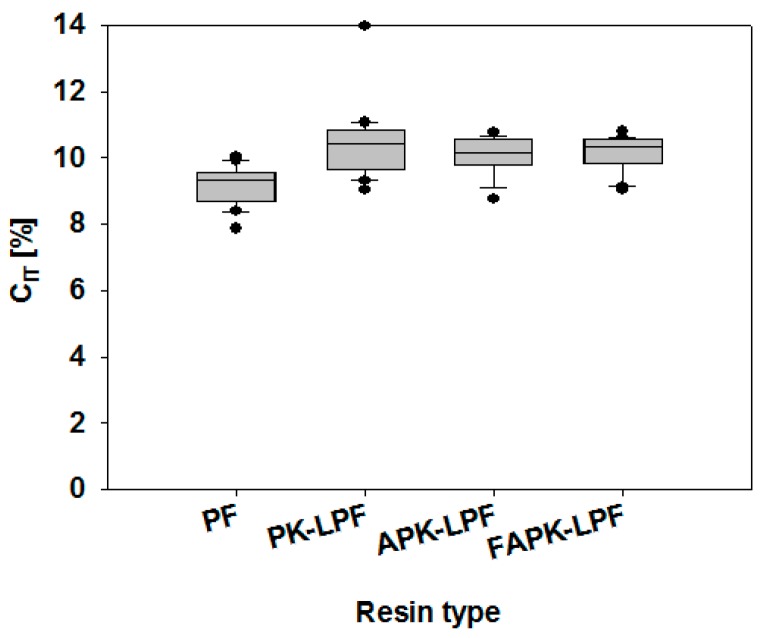
Indentation creep (C_IT_) of adhesive present in bonds prepared from the lignin-free reference material (PF) and PK-LPF, APK-LPF and FAPK-LPF resins.

Nanoindentation studies revealed how the replacement of phenol by non-modified or ammoxidized kraft lignin impact the mechanical properties of the different cured adhesive polymers. It has been found that both reduced elastic modulus and hardness of all tested resins (PK-, APK- and FAPK-LPF, Figure 7) were generally in the same range as reported for a wide variety of phenolic resins [38]. The obtained results were evaluated by non-parametric statistical tests using the R software package. Due to lack of normal distribution (Shapiro-Wilk Test) a Kruskal-Wallis Test (H-Test) and a pair-wise comparison of the individual groups by the Wilcoxon-Mann-Whitney Test (U-Test) were performed using a 0.05 level of significance. A comparison of the cured adhesive polymers prepared in this study revealed only marginal differences in terms of absolute values. Nevertheless, these differences were found to be statistically significant when comparing the adhesive present in bonds obtained from the PF reference material and that prepared from the resins containing the differently modified kraft lignins. The lignin-containing adhesive polymers showed higher reduced elastic modulus Er combined with lower hardness H, and a higher tendency to creep C_IT_ (Figure 7 and Figure 8). In contrast, the observed differences using the ABES bond strength development, which were tested in hot conditions as described previously, show that the lignin-containing adhesives no longer perform inferiorly at room temperature. As discussed in [38], a decreasing value of the hardness to elastic modulus (H/E—ratio) may be related to increasing toughness of the adhesive polymer. For this reason, the lignin-containing adhesive polymers may be described to have reduced brittleness compared to the lignin-free reference. Generally speaking, it may be concluded that the substitution of phenol by lignin has a marginal positive effect on the mechanical properties of the LPF polymer. Statistical analysis also revealed a significantly higher hardness, which may be related to yield strength of the adhesive [38] for the adhesive bonds obtained from FAPK-LPF compared to APK-LPF which could be explained by the significantly lower average molecular weight of FAPK and the stiffer network formed by cross-linking with the intermediary *N*,*N*,*N*-tris(methylol) trimethylene triamine.

## 4. Conclusions

The conducted study confirms that ammoxidation of pine kraft lignin increases its molecular weight considerably by depolymerization, introduction of functional groups and subsequent condensation reactions mediated by ammonia. The distinct gain in average molecular weight—most pronounced for APK—leads to significant phenol formaldehyde (PF) network extension during cooking of ammoxidized pine kraft lignin (APK) and Fenton-activated and subsequently ammoxidized pine kraft lignin (FAPK)−lignin phenol formaldehyde (LPF) resoles as reflected by the faster increase of viscosity compared to the PF reference or the PK-LPF resin. Fenton-type oxidation of lignin prior to ammoxidation accelerates the cooking process even more, likely through intermediary formation of the trivalent cross-linker *N*,*N*,*N*-tris (methylol) trimethylene triamine triggered by saponification of the higher fraction of ammonium salts present in FAPK and the reaction of the released ammonia with formaldehyde. Even though replacement of phenol by 40 wt % of (ammoxidized) lignin generally improves the curing behaviour of the resulting resins in terms of B-time (declining in the order PF > PK-LPF > APK-LPF > FAPK-LPF), this acceleration could no longer be observed in the presence of wood, where a significantly delayed wood-adhesive bond formation in terms of tensile shear strength development and final tensile shear strength (tested in hot state) were evident for PK-LPF and even more so for APK-LPF and FAPK-LPF. Low indentation creep of the cured lignin-free PF reference adhesive cannot be reached at such a high level of phenol replacement. However, all lignin-containing adhesive polymers show reduced brittleness compared to PF, as a result of higher elastic modulus and reduced hardness values.

## Figures and Tables

**Table 1:** Sample codes.

	Acronym	Sample Name
Lignin	PK	Pine kraft lignin
	APK	Ammoxidized pine kraft lignin
	FAPK	Fenton-activated and subsequently ammoxidized pine kraft lignin
Resin	PF	Phenol formaldehyde resin
	PK-LPF	(Pine kraft lignin) phenol formaldehyde resin
	APK-LPF	(Ammoxidized pine kraft lignin) phenol formaldehyde resin
	FAPK-LPF	(Fenton-activated and subsequently ammoxidized pine kraft lignin) phenol formaldehyde resin

**Table 2:** Molecular weight distributions and sugar content of pine kraft (PK) lignin and of the two derivatives obtained by ammoxidation without (APK) and with prior Fenton-type oxidation (FAPK).

	Molecular weight average * (Da)				Monosaccharide content (µm·mg^−1^ TS)			
Lignin type	*M*_n_ (Da)	*M*_w_ (Da)	*M*_z_ **	*M*_w_/*M*_n_	Arabinose	Fucose	Xylose	Galactose
PK	174	3971	17,783	23	2.06	3.73	5.74	4.47
APK	478	13,859	40,853	29	1.94	5.98	5.01	3.68
FAPK	474	6495	18,578	14	2.00	7.93	2.56	2.89

* Calculated molar masses based on PSS standards (Da). ** Mz: Size average molecular weight.

**Table 3:** Results of ultimate analysis (ash-free matter), H/C, N/C, O/C atomic ratios and aromaticity indices of pine kraft (PK) lignin and its two derivatives APK (ammoxidation) and FAPK (Fenton oxidation and ammoxidation) as calculated from the integral ratio of protons attached to aromatic and aliphatic moieties (^1^H-NMR) or the Rentrop function.

Sample	C	H	O	N	H/C	N/C	O/C	aromaticity	
	(wt %)				(at. ratio)			^1^H-NMR	*f*_a_
PK	62.86	6.04	29.93	1.17	1.1449	0.0160	0.3574	1.27	0.852
APK	54.71	5.71	33.53	6.06	1.2436	0.0950	0.4601	1.65	0.945
FAPK	55.47	5.61	32.84	6.08	1.2051	0.0940	0.4444	1.39	0.943
FAPK	55.47	5.61	32.84	6.08	1.2051	0.0940	0.4444	1.39	0.943

**Table 4:** Contents of total, aliphatic and phenolic hydroxyl groups as quantified by ^31^P-NMR spectroscopy after derivatization of the samples with 2-chloro-4,4,5,5-tetramethyl-1,3,2-dioxa-phospholane and contents of selected C–, O–, and N–structural elements (at %) as suggested by deconvolution and quantification of X-ray photoelectron spectra.

	^31^P-NMR spectroscopy			X-ray photoelectron spectroscopy						
Sample	OH_AL_	OH_AR_	OH_tot_	C–O–CC–OH	C–N	N–R_3_	N–CR=O	O=CR_2_	R–O–C	O–CO–R
	mmol·g^−1^			at %						
PK	2.28	2.90	5.19	25.6 ± 1.3	0.4 ± 0.0	0.4 ± 0.1	0.6 ± 0.0	4.8 ± 0.3	16.2 ± 0.1	2.5 ± 0.1
FAPK	0.83	1.16	1.99	23.2 ± 0.6	3.8 ± 0.3	3.4 ± 0.1	0.8 ± 0.0	6.5 ± 0.9	14.4 ± 0.4	1.7 ± 1.3
APK	0.60	0.99	1.59	7.5 ± 0.2	4.5 ± 0.3	3.7 ± 0.3	1.7 ± 0.4	24.2 ± 0.7	10.3 ± 1.6	3.2 ± 0.2

**Table 5:** Selected properties of the prepared resole resins.

Resin type	Viscosity (mPa·s)	Final pH	Solid content (wt %)	Free HCHO (wt %)
PF	1020	10.2	43.2	2.0
PK-LPF	847	10.4	43.3	9.2
APK-LPF	901	10.2	42.1	8.1
FAPK-LPF	1050	10.6	43.7	8.8

**Table 6:** B-time of the prepared resole resins at 100 °C.

Resin type	PF	PK-LPF	APK-LPF	FAPK-LPF
B-time	7 min 38 s	5 min 37 s	5 min 6 s	4 min 50 s

**Table 7:** Curing properties of the prepared resole resins as investigated by DSC.

Resins	*T*_onset_ (°C)	*T*_peak_ (°C)	*T*_endset_ (°C)	Heat of cure reaction (µVs/mg)		
				Peak A	Peak B	Total
PF	131.4	135.7	144.3	780.8	--	780.8
PK-LPF	177.2	182.1	191.6	504.1	279.3	783.4
APK-LPF	173.6	180.2	189.8	417.5	255.5	673.0
FAPK-LPF	171.7	179.3	189.3	408.1	234.3	642.4
